# The Prevalence and Pathophysiology of Chemical Sense Disorder Caused by the Novel Coronavirus

**DOI:** 10.3389/fpubh.2022.839182

**Published:** 2022-06-06

**Authors:** Sareesh Naduvil Narayanan, Pooja Shivappa, Sreeshma Padiyath, Anand Bhaskar, Yan Wa Li, Tarig Hakim Merghani

**Affiliations:** ^1^Department of Physiology, Ras Al Khaimah College of Medical Sciences, Ras Al Khaimah Medical and Health Sciences University, Ras Al Khaimah, United Arab Emirates; ^2^Department of Basic Sciences, Ras Al Khaimah College of Medical Sciences, Ras Al Khaimah Medical and Health Sciences University, Ras Al Khaimah, United Arab Emirates; ^3^Independent Microbiology Researcher, Ras Al Khaimah, United Arab Emirates; ^4^Department of Biomedical Sciences, Faculty of Medicine, Macau University of Science and Technology, Taipa, Macau SAR, China

**Keywords:** novel coronavirus disease (COVID-19), angiotensin-converting enzyme 2 receptor (ACE-2), chemical senses, olfactory dysfunction, taste dysfunction

## Abstract

Emerging viral infections are a ceaseless challenge and remain a global public health concern. The world has not yet come back to normal from the devastating effects of the highly contagious and pathogenic novel coronavirus, or Severe Acute Respiratory Syndrome Coronavirus 2 (SARS-CoV-2). Olfactory and taste dysfunction is common in patients infected by the novel coronavirus. In light of the emergence of different coronavirus variants, it is important to update the prevalence and pathophysiology of these side effects. In this review, articles published on the prevalence of olfactory and taste dysfunction from coronavirus disease (COVID-19) and their possible pathophysiologic mechanisms have been reviewed and reported. The modulatory role of different SARS-CoV-2 variants on the chemical senses is then described. The clinical relevance of chemical sense disorder and its long-term morbidity and management is also discussed.

## Introduction

Emerging viral infections are a ceaseless challenge and remain a global public health concern. Coronaviruses (CoVs) are a highly diverse family of RNA viruses that can infect humans, wild or domestic mammals, and birds (1). The novel coronavirus, Severe Acute Respiratory Syndrome Coronavirus 2 (SARS-CoV-2), is an enveloped, positive-sense single-stranded RNA (ssRNA) virus with a genomic size of 29.9 kb that is contagious and pathogenic (2). The coronavirus disease (COVID-19) caused by SARS-CoV-2 has spread globally and is still spreading exponentially. On 11 March 2020, the World Health Organization (WHO) declared COVID-19 a pandemic. Of the approximately 359 million confirmed COVID-19 cases worldwide, a large number had nervous system complications, including chemosensory dysfunction, visual impairment, distal weakness, headache, dizziness, insomnia, and delirium (3). Particular concerns have been raised about chemosensory dysfunction from COVID-19 infection because of its link with a wide range of neurodegenerative diseases (4).

Chemosensation involves the transduction of a chemical stimulus from the environment into a neurological signal interpreted by the organism. Chemosensory systems directly interact with environmental chemical cues and regulate behaviors essential for survival (5). In humans, olfaction represents an evolutionarily critical physiologic system and plays a significant role in mood, emotion, pleasure sensation, memory, and many other processes of the central nervous system (6). The sense of taste is responsible for the detection and ingestion of food to satisfy energy requirements in a healthy or disease state. Altered taste perception can affect the patient's appetite, body weight, and psychological wellbeing, thereby reducing their quality of life (7). Loss of taste perception may also lead to malnutrition, which is usually one of the most frequent causes of morbidity and mortality in patients with chronic diseases. Although there have been studies on olfactory and taste dysfunction caused by COVID-19, it is important to update the prevalence of this dysfunction and its possible pathophysiology. Because SARS-CoV-2 is mutating at a much faster rate, the impact of these variants on chemosensory function is poorly understood and has not been well documented. This review focuses on articles published on the prevalence of COVID-19 olfactory and taste dysfunction, attempts to identify the possible pathophysiological mechanisms for these adverse events, and defines the modulatory roles of different coronavirus variants on the chemical senses. Finally, the clinical relevance of chemical sense disorder, its long-term morbidity and the management of patients with COVID-19 who present with chemical sense disorder is discussed.

## Methods

Major electronic databases such as MEDLINE/PubMed/SCOPUS/Web of Science/Embase and Google Scholar were searched for this study. Key search terms included: “COVID-19,” “Novel coronavirus,” “Novel coronavirus” AND “COVID-19” “Coronavirus disease”/“SARS-CoV-2”/“COVID-19” AND “ACE2 receptor”/“Neurological symptoms”/“Olfaction”/“Olfactory epithelium”/“ACE2 expression on olfactory epithelium”/“Anosmia”/“Anosmia prevalence”/“Anosmia mechanisms”/“Gustation”/“Taste buds”/“ACE2 expression in oral mucosa”/“Ageusia”/“Dysgeusia”/“Dysgeusia prevalence”/“Dysgeusia mechanisms”, “Novel coronavirus”/“SARS-CoV-2”/“COVID-19” AND “Mutation”/“Variants,” “Novel coronavirus”/“SARS-CoV-2”/“COVID-19” AND “Chemical sense dysfunction significance”/“Long-term morbidity”/“Management”. Several publicly available sources from regulatory authorities such as the World Health Organization (WHO) and the U.S. Centers for Disease Control and Prevention (CDC) were also used if they contained information about the review. All titles and abstracts identified *via* the MEDLINE/PubMed/SCOPUS search that were relevant to the topic of this review were vetted. Those works that were not relevant to the specific topic of this review were excluded. Every article that discussed any of the above endpoints/parameters was retained. Non-English articles were excluded. The full texts of the articles that meet our inclusion criteria were obtained, reviewed, analyzed, and summarized. Chemical sensory dysfunction prevalence data from all reports were pooled and the mean prevalence for Europe, the Americas, the Middle East, Asia, and Africa was calculated.

### Olfactory Dysfunction Caused by COVID-19

#### Physiology of Olfaction, ACE2 Expression in the Olfactory Epithelium, and Infection Mechanism

**Figure 1:**
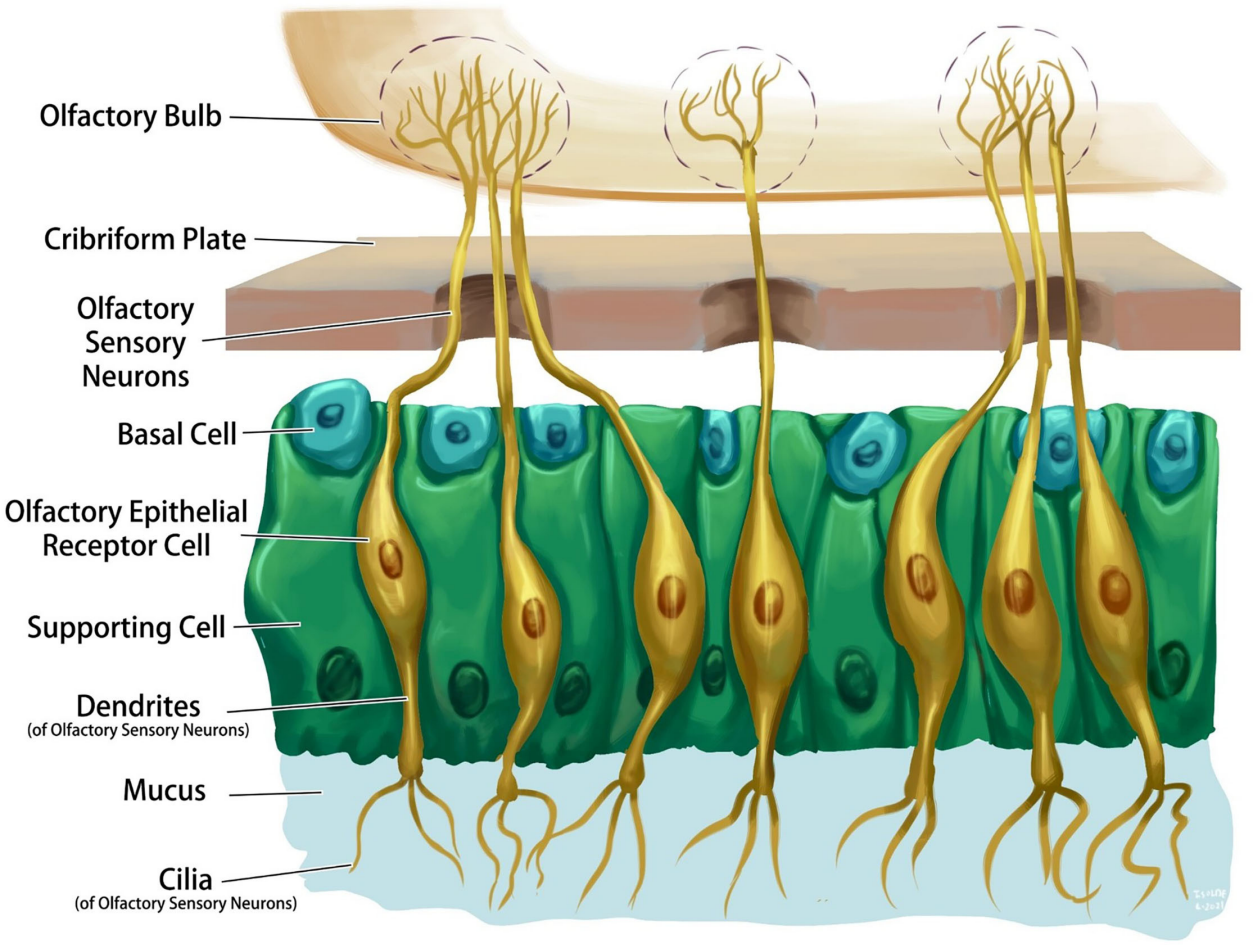
Schematic representation of normal olfactory epithelium and its associated structures.

The olfactory system is one of the few central nervous system structures that has direct access to the external environment. Olfactory sensory neurons are bipolar cells that connect the receptors with the olfactory bulb on the ventral aspect of the frontal lobe through the cribriform plate of the ethmoid bone. The olfactory epithelium consists of four primary cell types: olfactory sensory neurons, supporting cells, basal cells, and brush cells (Figure 1). The chemical interactions between volatile compounds and the chemoreceptors present on olfactory sensory neurons result in the sense of smell. Inhaled molecules enter the nose and reach the olfactory epithelium, where they dissolve into mucus. Some proteins keep the odorant molecules soluble in the mucus and transport them to the olfactory receptors. Receptor stimulation initiates electric potentials that transmit through the olfactory nerve to the olfactory bulb, then to the olfactory cortex in the temporal lobe, and to other associated brain structures (6). Olfactory projections help in the formation of long-term memories, emotional responses, and visceral responses *via* its diffused connections through the reticular formation, hippocampus, and amygdala (8).

The nasal epithelium is the first point of contact of SARS-CoV-2 with the human body. Reports demonstrate an age-dependent expression of ACE2 on the nasal epithelium (9). Airway epithelial cells contain motile cilia, that is the primary location where the ACE2 receptor protein is localized, and this seems to be the initial subcellular site of SARS-CoV-2 viral entry during host respiratory transmission (10). Immunolabeling for ACE2 in sagittal sections of the human nose revealed evidence of the presence of ACE2 in the olfactory epithelium and the respiratory epithelium of the nasal conchae, nasal septum, paranasal sinuses, and olfactory bulb. ACE2 was also detected in sustentacular cells, glandular cells of the olfactory epithelium, as well as in the basal cells, glandular cells, and epithelial cells of the respiratory epithelium. ACE2 is also expressed on the olfactory bulb but not in mature or immature olfactory receptor neurons and basal cells of the olfactory epithelium (11).

The entry of the virus into the host is the primary stage of viral infection. The receptor-binding domain of the spike protein on the surface of the COVID-19 virus binds to the top of sub-domain I of the angiotensin-converting enzyme 2 (ACE2) receptor on the host cell to enter it. The ACE2 receptor can mediate the entry of the virus into the host either *via* cell membrane fusion or macrophage phagocytosis. Transmembrane proteinases such as TNF-converting enzyme, transmembrane protease serine-2 (TMPRSS2), disintegrin and metallopeptidase domain 17 (ADAM17), and other proteins such as clathrin and vimentin promote the binding and membrane fusion process. Viral RNA is then released into the cytoplasm. The COVID-19 virus then translates its RNA replicase (RNA-dependent RNA polymerase) and makes an RNA replicase–transcriptase complex using its RNA template. During replication, the RNA replicase–transcriptase complex forms a complex strand of negative RNA that will be translated to form the viral structural proteins. New viral particles form in the cytoplasm of the infected cell along with its structural proteins and RNA, which are released from the host cell to infect the surrounding cells (12).

### Prevalence of Olfactory Dysfunction in Patients With COVID-19

#### Europe

A cross-sectional study (*n* = 59) performed in Italy showed that 33.9% of COVID-19 patients had olfactory dysfunction (13). Vaira et al. (14) conducted a retrospective study in Italy on 138 patients with COVID-19 and reported that 50% of patients had both olfactory and taste dysfunction, while 18.8% had isolated olfactory dysfunction. In another study, Vaira et al. (15) reported that 75.8% of COVID-19 patients self-reported olfactory dysfunction. In a multicenter study carried out in European countries (Belgium, France, Spain, and Italy), 85.6% of 417 patients had olfactory dysfunction (16). Menni et al. (17) conducted a cross-sectional study in the United Kingdom, reporting that 59.41% of 579 patients had olfactory dysfunction. Another cross-sectional study (*n* = 202) by Spinato et al. (18) in the United Kingdom found that 64.4% of COVID-19 patients had olfactory dysfunction. An observational cohort study by Makaronidis et al. (19) observed a loss of smell in 93.4% of patients with SARS-CoV-2 antibodies. Klopfenstein et al. (20) performed a retrospective evaluation of olfactory dysfunction among 114 patients with COVID-19, observing that 47% of patients had olfactory dysfunction. Tudrej et al. (21) conducted a cross-sectional study of 816 patients, of whom 19.1% had olfactory dysfunction. In Spain, Beltran-Corbellini et al. (22) carried out a case–control study including 79 patients with COVID-19 documented an olfactory dysfunction prevalence of 31.65%. A case–control study of COVID-19 patients (*n* = 355) found subjective hyposmia in 64.1% of cases (23). Villarreal et al. (24) conducted a descriptive observational study of 256 healthcare workers with COVID-19 that reported olfactory dysfunction in 68% of subjects. A study from Turkey recorded a prevalence of anosmia of 51.2% (25), while another study from Turkey documented an anosmia prevalence of 31.8% (26). Özçelik Korkmaz et al. (27) reported a hyposmia/anosmia prevalence of 37.9% (27). A cross-sectional study including 1,942 patients with COVID-19 from Poland observed olfactory dysfunction in 49.2% (28). Finally, a study from Switzerland documented an olfactory dysfunction in 61.2% of COVID-19 patients (29).

#### Americas

Yan et al. (30) carried out a cross-sectional study on 59 patients, of whom 68% had olfactory dysfunction. Dawson et al. (31) documented that of the 90 participants enrolled in their study, 62% reported anosmia. A study from Georgia, United States, observed anosmia in 51% of COVID-19 positive cases (32), while another study from the United States recorded a loss of smell in 30% of patients (33). Laws et al. (34) performed a cohort study of pediatric patients and detected a loss of smell in 32% of COVID-19-positive cases (34). A cross-sectional study from Canada reported an anosmia prevalence of 41.1% (35), while a prospective survey from Brazil demonstrated a prevalence of olfactory disturbances of 82.4% in COVID-19 patients (36). Another study among Latin American ethnic patients reported olfactory dysfunction in 81.9%, of whom 67.5% had a partial loss of smell and 14.4% had a total loss of smell (37).

#### Middle East

Bagheri et al. (38) performed a cross-sectional survey of 10,069 Iranians, reporting that 60.90% of responders had anosmia. In another case–control study (*n* = 60), Moein et al. (39) documented that 98.33% of COVID-19-positive patients had olfactory dysfunction. Alshami et al. (40) studied 128 COVID-19 patients at a quarantine facility in Saudi Arabia and recorded a prevalence of olfactory dysfunction of 47.5%. Al-Rawi et al. (41) carried out telephone interviews with 500 COVID-19 patients, of whom 44% had anosmia. A retrospective study from Qatar documented anosmia in 13.47% of COVID-19-positive cases (42), whereas a case series study from Israel (*n* = 140) reported impaired smell in 38.3% of COVID-19 patients (43).

#### Asia

A cross-sectional study by Jain et al. (44) (*n* = 410) found that 21.1% of COVID-19 patients had anosmia. Another cross-sectional study (*n* = 230) by Rajkumar et al. (45) documented anosmia prevalence of 41.3%. Bidkar et al. (46) evaluated the odor sensing of 76 COVID-19 patients, finding that 81.6% were anosmic. Panda et al. (47) performed a study on 225 COVID-19 patients, of whom 12.5% self-reported anosmia. Similarly, Krishnasamy et al. (48) reported that 9.4% of 1,263 COVID-19 patients self-reported anosmia. In another case–control study on 261 patients, Dev et al. (49) observed that 21.1% had anosmia during the time that they were COVID-19 positive. In a prospective observational study conducted by Yadav et al. (50), 18.4% of patients had anosmia. Similarly, Thakur et al. (51) carried out a prospective study on 250 COVID-19-positive patients, of whom 71.6% had anosmia.

A retrospective cross-sectional study by Tham et al. (52) observed that 15.7% of COVID-19 patients had an olfactory loss. Kim et al. (53) performed a study on 213 individuals with COVID-19 and found that 39.5% had hyposmia. Mao et al. (54) in their retrospective multicenter study (*n* = 214, hospitalized patients) found that 5.1% of patients had an impaired sense of smell. In an observational cohort study on 199 patients, Noh et al. (55) documented an anosmia prevalence of 26.1%. Song et al. (56) performed a retrospective study on 1,206 COVID-19 patients, observing that 11.4% had lost their sense of smell.

#### Africa

An observational study from Tunisia reported smell impairment in 37.9% of patients. Of these, 65.3% had anosmia, 26.6% patients had parosmia, and 10.9% patients had olfactory hallucinations (57). A retrospective cohort study from Nigeria documented anosmia in 1.6% of COVID-19 patients (58), while another retrospective double center study from Somalia reported anosmia in 40% of patients (59). Olfactory dysfunction prevalence data from all of these individual studies were pooled and mean olfactory dysfunction prevalence was calculated (Table 1).

**Table 1:** Prevalence of olfactory dysfunction.

**Region/Country**	**References**	**Study design**	**Sample size**	**Prevalence (%)**
**Europe**				
Italy	Giocomelli et al. (13)	Cross sectional study	59	33.9
Italy	Vaira et al. (14)	Prospective study	138	68.8
Italy	Vaira et al. (15)	Prospective study	106	75.8
Belgium, France, Spain, Italy	Lechien et al. (16)	Multicenter study	417	85.6
UK	Menni et al. (17)	Community survey	1,702	59.41
UK	Spinato et al. (18)	Prevalence study	202	64.4
UK	Makaronidis et al. (19)	Observational cohort study	567	93.4
France	Klopfenstein et al. (20)	Retrospective observational study	114	47
France	Tudrej et al. (21)	Cross sectional study	816	19.1
Spain	Beltran-Corbellini et al. (22)	Case- control study	119	31.65
Spain	Martin-Sanz et al. (23)	Prospective study	355	64.1
Spain	Villarreal et al. (24)	Observational study	256	68
Turkey	Sakalli et al. (25)	Cross sectional study	172	51.2
Turkey	Salepsci et al. (26)	Cross sectional study	223	31.8
Turkey	Özçelik Korkmaz et al. (27)	Prospective observational cohort study	116	37.9
Poland	Sierpiński et al. (28)	Cross sectional study	1,942	49.2
Switzerland	Speth et al. (29)	Prospective cross-sectional study	103	61.2
Prevalence of olfactory dysfunction in Europe				Mean - 55.44%
**Americas**				
USA	Yan et al. (30)	Cross sectional study	1,480	68
USA	Dawson et al. (31)	Household study	90	62
USA	Kempker et al. (32)	Screening	283	51
USA	Smith et al. (33)	Retrospective cohort	240	30
USA	Laws et al. (34)	Cohort study	188	32
Canada	Lee et al. (35)	Cross sectional study	127	41.1
Brazil	Brandão Neto et al. (36)	Prospective survey	655	82.4
Latin America	Chiesa-Estomba et al. (37)	Cross sectional study	542	81.9
Prevalence of olfactory dysfunction in Americas				Mean - 56.05%
**Middle East**				
Iran	Bagheri et al. (38)	Cross sectional study	10,069	60.90
Iran	Moein et al. (39)	Case control study	120	98.33
Saudi Arabia	Alshami et al. (40)	Cross-sectional study	128	47.5
UAE	Al-Rawi et al. (41)	Cross-sectional study	500	44
Qatar	Al-Ani and Acharya (42)	Retrospective study	141	13.47
Israel	Biadsee et al. (43)	Case series study	128	38.3
Prevalence of olfactory dysfunction in the Middle East				Mean - 50.42%
**Asia**				
India	Jain et al. (44)	Cross sectional study	410	21.1
India	Rajkumar et al. (45)	Cross sectional study	230	41.3
India	Bidkar et al. (46)	Cross sectional study	836	81.6
India	Panda et al. (47)	Prospective cohort study	225	12.5
India	Krishnasamy et al. (48)	Cross sectional study	1,263	9.4
India	Dev et al. (49)	Case control study	110	21.1
India	Yadav et al. (50)	Prospective observational study	152	18.4
India	Thakur et al. (51)	Prospective study	250	71.6
Singapore	Tham et al. (52)	Retrospective cross sectional study	1,065	15.7
Republic of Korea	Kim et al. (53)	Cross sectional study	172	39.5
China	Mao et al. (54)	Observational study	214	5.1
Republic of Korea	Noh et al. (55)	Observational cohort study	199	26.1
China	Song et al. (56)	Retrospective study	1,172	11.4
Prevalence of olfactory dysfunction in Asia				Mean - 28.83%
**Africa**				
Tunisia	Kacem et al. (57)	Retrospective observational study	646	37.9
Nigeria	Elimian et al. (58)	Retrospective cohort study	10,517	1.6
Somalia	Farah Yusuf Mohamud et al. (59)	Retrospective study	60	40
Prevalence of olfactory dysfunction in Africa				Mean - 26.50%
Prevalence of olfactory dysfunction (Europe, Americas, Middle East, Asia and Africa)				Pooled Mean - 45.69%, 95% CI (38.35, 53.04)

### Gustatory Dysfunction Caused by COVID-19

#### Physiology of Gustation and ACE2 Expression in the Gustatory Apparatus

**Figure 2:**
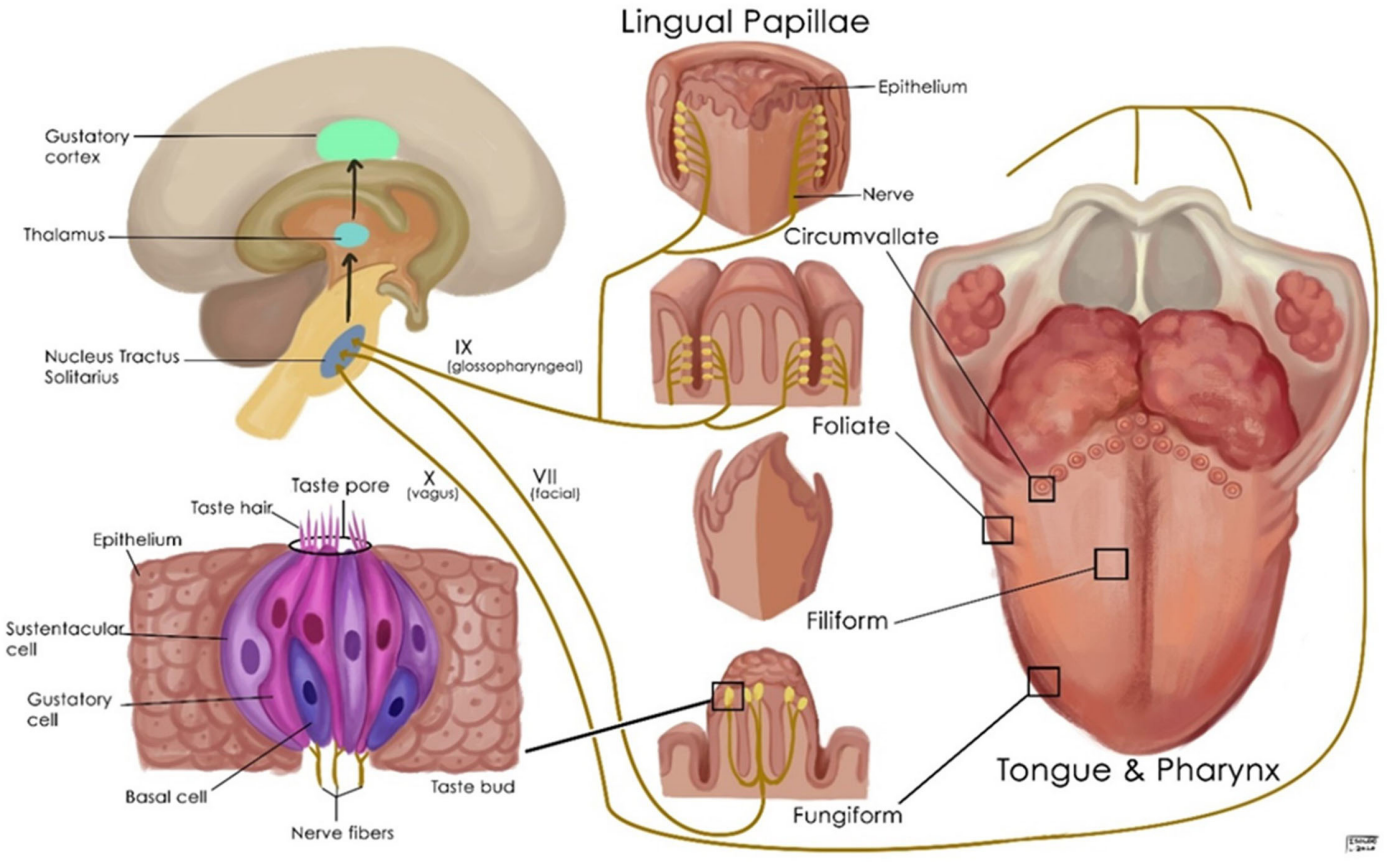
Schematic representation of the normal gustatory apparatus, its nerve supply, and its associated structures.

The perception of flavors is complex and involves the senses of taste, smell, and chemesthesis. Taste buds are clusters of epithelial cells that form compact, columnar pseudostratified islands distributed on the tongue, palate, and to a lesser extent the epiglottis, pharynx, and larynx (Figure 2). Taste buds in all regions respond to sweet, salty, sour, bitter, and umami, but there may be differences in their sensitivities to different tastes (60). There are four types of taste bud cells. Type I taste cells are the glia of the taste bud and play a role in detecting salty tastes. Type II taste cells are responsible for the detection of sweet, umami, and bitter compounds. Type III taste cells are thought to detect sour tastes, and type IV cells are basal cells that are presumably undifferentiated, and their significance is unknown (60). G-protein-coupled receptors (GPCRs) and channel-type receptors are expressed in taste bud cells, and their activation creates a taste sensation. Taste sensations are carried by cranial nerves VII, IX, and X to the nucleus of the tractus solitarius, then to the thalamus, and terminates in the antero-inferior part of sensorimotor cortex and insula (Figure 2). Taste information is also relayed to numerous hypothalamic nuclei and the limbic system (61).

ACE2 is expressed by the basal layer of the non-keratinizing squamous epithelium of the oral mucosa and is not found on the surface of the epithelium (62). ACE2 expression is significantly higher at the oral tongue compared with the base of the tongue and the mouth floor gingival and buccal tissues (63). ACE2 is expressed in the nuclei and cytoplasm of the spinous and basal cell layers of the dorsum of the tongue (64). TMPRSS2 is primarily expressed by the cell membrane of the horny layer of the tongue. Furin is expressed in the spinous-basal cell layers (64). Human fungiform papillae taste cells express ACE2, TMPRSS2, and furin. While ACE2 is seen in the nucleus, TMPRSS2 is weakly expressed and furin intensely expressed in the cytoplasm (63, 64).

### Prevalence of Gustatory Dysfunction in Patients With COVID-19

#### Europe

A multicenter European study on mild-to-moderate COVID patients reported a prevalence of gustatory dysfunction of 88% (16). The prevalence of taste dysfunction was 54.2% (65) and 56.4% (66) in two other European cohorts of mild-to-moderate COVID-19 patients. Vaira et al. (14) studied gustatory dysfunction in 138 patients with COVID-19, observing that 15.9% had isolated taste dysfunction and 50% had both olfactory and taste dysfunction. A multicenter prospective study from Italy by Vaira et al. (15) reported gustatory impairment in 65.6% of COVID-19 patients. Another multicenter study carried out by Vaira et al. (67) found that the prevalence of gustatory disturbance in COVID-19 patients was 67.8%, with 10.4% having ageusia and 34.5% having mild, moderate, or severe hypogeusia. An observational multicenter study reported a prevalence of taste disorder of 59.2% (68), while a study from Northern Italy found partial taste loss in 26.5% of home-quarantined COVID-19 patients and complete taste loss in 62.9% (69). Another study by Paderno et al. (70) in Brescia, Italy, observed overall gustatory dysfunction in 63% of cases, while taste abnormalities were more prevalent among home quarantined COVID-19 patients with less severe disease (72%) than hospitalized COVID-19 patients (52%). In a prospective study, in Bologna, Italy, Paolo et al. (71) found that all COVID-19 patients had dysgeusia (71).

A multicenter study from Madrid, Spain, showed that taste abnormalities were present in 90.3% of COVID-19 patients and 45.2% had ageusia. The onset of a taste disorder was acute and mostly seen in younger patients, but they recovered from it within 1–2 weeks (22). Also in Madrid, Martin-Sanz et al. (23) observed hypogeusia in 53% of COVID-19 positive health care workers (23). An observational study in Madrid on health care workers with COVID-19 reported altered taste in 70% (24) and the mean recovery time was 11 days, although in 26% of the health care workers the symptoms persisted for longer than 1 month. Another multicenter study from Spain reported a prevalence of taste abnormalities among COVID-19 patients of 52.2% (72). Researchers in Istanbul, Turkey, observed that 47.1% of COVID-19 patients lost their sense of taste (25). A cross-sectional study from Istanbul, Turkey, documented that 34.5% of COVID-19 patients had ageusia (26), while another prospective observational cohort study from Sakarya, Turkey, reported hypogeusia and ageusia in 41.3% of COVID-19 patients (27).

A retrospective cross-sectional study from Lyon, France, observed ageusia and hypogeusia in 23% of COVID-19 patients (21), while a study on COVID-19 patients from Strasbourg, France, documented an ageusia prevalence of 35.1% (73).

A cross-sectional study from Poland observed that 47.5% of patients self-reported taste dysfunction and that taste dysfunction was more frequent in women (28). Another prospective cross-sectional study from Switzerland documented a loss of taste in 65% of COVID-19 patients (29). In a seroprevalence study from the United Kingdom, loss of taste was seen in 90.2% of patients with SARS-CoV-2 antibodies (19). In a study conducted in Belgium, Le Bon et al. (74) observed dysgeusia in 7% of patients 5 weeks after COVID-19. Another cross-sectional study from the Faroe Islands showed loss of taste in 40.6% of COVID-19 patients during the acute infection phase, which improved to 16.1% during follow-up (75).

#### Americas

In a study from La Jolla, United States Yan et al. (30) documented impaired taste in 71% of COVID-19 patients. In another study from the United States, 57% of COVID-19 patients reported the loss of taste sensation and 33% had a complete loss of taste (31). A screening study from Atlanta, Georgia, United States, reported that 53% of health care workers with COVID-19 lost their sense of taste (32). Another retrospective cohort study from New Haven, Connecticut, United States, reported an ageusia prevalence of 34.2% (33). Laws et al. (34) observed that 21% of children with COVID-19 lost their sense of taste. Lee et al. (35) carried out a cross-sectional study in Canada documented a prevalence of 46.4% for dysgeusia and ageusia in patients with COVID-19 (35). A prospective survey from Brazil found general taste loss in 76.2% of COVID-19 patients and specific taste loss (sweet, sour, salty, and bitter) in 52.2% (36). A cross-sectional study on Latin-American COVID-19 patients from Spain, Uruguay, Venezuela, and Argentina reported a taste disorder prevalence of 61.4% (37).

#### Middle East

Al-Ani and Acharya (42) reported that ageusia was seen in 19.8% of COVID-19 patients in Qatar (42), while taste impairment was seen in 16.3% of COVID-19 patients in Iran (76). A case–control study from Iran observed taste loss in 24% of COVID-19 patients (39). A case series study from Israel documented that 32.8% of COVID-19 patients reported an impaired sense of taste, most of whom were women (43).

#### Asia

A study from Hong Kong showed taste dysfunction in 43.4% of COVID-19 patients and found no correlation between taste dysfunction and viral load (77). According to a report by Lv et al. (78) in Wuhan, China, taste impairment was seen in 11.73% of COVID-19 patients. In a retrospective analysis from Japan, taste dysfunction was seen in 56.3% of COVID-19 patients (79). A study from Faridabad, India, reported a prevalence of taste dysfunction in 22.4% of COVID-19 patients (44). Bidkar et al. (46) performed a cross-sectional study in Nagpur, India, observed ageusia in 84.2% of COVID-19 patients. In a study carried out by Panda et al. (47), dysgeusia was seen in 17.33% of COVID-19 patients in New Delhi, India. Rajkumar et al. (45) reported taste dysfunction in 10.9% of COVID-19 patients in Chennai, India, while a prospective observational study from Patiala, India, reported gustatory dysfunction in 13.15% of patients (50). Makda et al. (80) performed a cross-sectional study in Pakistan that reported taste impairment in 7.8% of COVID-19 patients. According to a retrospective cross-sectional, study by Tham et al. (52), taste disorder was seen in 8.5% of COVID-19 patients in Singapore (52), while a study from Malaysia showed gustatory dysfunction in 23.4% of COVID-19 patients (81).

#### Africa

Kacem et al. (57) performed a retrospective observational study on COVID-19 patients in Tunisia reported a taste impairment prevalence of 36.8%. A study carried out by Elimian et al. (58) in Nigeria observed ageusia in 1.2% of COVID-19 patients, while another retrospective study from Somalia found ageusia in 28.3% of COVID-19 patients (59). Gustatory dysfunction prevalence data from these reports were pooled, and a mean gustatory dysfunction prevalence was calculated (Table 2).

**Table 2:** Prevalence of gustatory dysfunction.

**Region/Country**	**References**	**Study design**	**Sample size**	**Prevalence (%)**
**Europe**				
Italy, Belgium, France	Lechien et al. (16)	Multicenter study	417	88
Multicenter	Lechien et al. (65)	Cross sectional study	1,420	54.2
Multicenter	Lechien et al. (66)	Cross sectional study	2,013	56.4
Italy	Vaira et al. (14)	Prospective study	138	65.9
Italy	Vaira et al. (15)	Prospective study	106	65.6
Italy	Vaira et al. (67)	Multicenter cohort study	345	67.8
Italy	Barillari et al. (68)	Observational study	249	59.2
Northern Italy	Paderno et al. (69)	Prospective cohort study	151	89.4
Italy	Paderno et al. (70)	Cross sectional study	508	63
Italy	Paolo et al. (71)	Prospective study	75	100
Spain	Beltrán-Corbellini et al. (22)	Case- control study	119	90.3
Spain	Martin-Sanz et al. (23)	Prospective study	355	53
Spain	Villarreal et al. (24)	Observational study	256	70
Spain	Izquierdo-Domínguez et al. (72)	Multicenter cross sectional study	846	52.2
Turkey	Sakalli et al. (25)	Cross sectional study	172	47.1
Turkey	Salepci et al. (26)	Cross sectional study	223	34.5
Turkey	Özçelik Korkmaz et al. (27)	Prospective observational cohort study	116	41.3
France	Tudrej et al. (21)	Cross sectional study	816	23
France	Renaud et al. (73)	Retrospective cross sectional study	97	35.1
Poland	Sierpiński et al. (28)	Cross sectional study	1,942	47.5
Switzerland	Speth et al. (29)	Prospective cross-sectional study	103	65
UK	Makaronidis et al. (19)	Observational cohort study	567	90.2
Belgium	Le Bon et al. (74)	Prospective cohort study	72	7
Faroe Island	Petersen et al. (75)	Cross sectional study	180	40.6
Prevalence of gustatory dysfunction in Europe				Mean - 58.60%
**Americas**				
USA	Yan et al. (30)	Cross sectional study	1,480	71
USA	Dawson et al. (31)	Household study	90	57
USA	Kempker et al. (32)	Screening	283	53
USA	Smith et al. (33)	Retrospective cohort	240	34.2
USA	Laws et al. (34)	Cohort study	188	21
Canada	Lee et al. (35)	Cross sectional study	127	46.4
Brazil	Brandão Neto et al. (36)	Prospective survey	655	76.2
Latin America	Chiesa-Estomba et al. (37)	Cross sectional study	542	61.4
Prevalence of gustatory dysfunction in Americas				Mean - 52.53%
**Middle East**				
Qatar	Al-Ani and Acharya (42)	Retrospective study	141	19.8
Iran	Jalessi et al. (76)	Prospective descriptive study	92	16.3
Iran	Moein et al. (39)	Case control study	120	24
Israel	Biadsee et al. (43)	Case series study	128	32.8
Prevalence of gustatory dysfunction in the Middle East				Mean - 23.23%
**Asia**				
Hong Kong	Cho et al. (77)	Prospective cross sectional cohort study	143	43.4
China	Lv et al. (78)	Retrospective cross sectional study	196	11.73
Japan	Nakanishi et al. (79)	Retrospective study	160	56.3
India	Jain et al. (44)	Cross sectional study	410	22.4
India	Bidkar et al. (46)	Cross sectional study	836	84.2
India	Panda et al. (47)	Prospective cohort study	225	17.3
India	Rajkumar et al. (45)	Cross sectional study	230	10.9
India	Yadav et al. (50)	Prospective observational study	152	13.15
Pakistan	Makda et al. (80)	Cross sectional study	114	7.8
Singapore	Tham et al. (52)	Retrospective cross sectional study	1,065	8.5
Malaysia	Ramasamy et al. (81)	Cross sectional study	145	23.4
Prevalence of gustatory dysfunction in Asia				Mean - 27.19%
**Africa**				
Tunisia	Kacem et al. (57)	Retrospective observational study	646	36.8
Nigeria	Elimian et al. (58)	Retrospective cohort study	10,517	1.2
Somalia	Farah Yusuf Mohamud et al. (59)	Retrospective study	60	28.3
Prevalence of gustatory dysfunction in Africa				Mean - 22.10%
Prevalence of gustatory dysfunction (Europe, Americas, Middle East, Asia and Africa)				Pooled Mean - 45.70%, 95% CI (38.33, 53.06)

### Pathophysiology of Olfactory and Gustatory Dysfunction Caused by COVID-19

#### Pathophysiological Mechanisms of Olfactory Dysfunction

Anosmia and hyposmia can result from disease processes, viral infections, environmental exposure, or aging. Olfactory dysfunction is highly suggestive of SARS-CoV-2 infection, even among asymptomatic subjects (48). The sense of smell is acutely lost in COVID-19 patients. There may be multiple reasons for decreased or lost smell sensitivity in humans. There is a consensus that smell loss from COVID-19 is not the result of virus-induced nasal congestion. Different mechanisms leading to olfactory dysfunction are discussed in brief in the proceeding sections.

##### Role of Inflammation of Nasal Mucosa

Many viruses (rhinovirus, parainfluenza, Epstein–Barr virus, and some coronaviruses) cause inflammation within the olfactory epithelium that results in olfactory dysfunction. However, the CT/MRI scans of COVID-19 patients with anosmia show inflamed olfactory clefts without nasal obstruction or rhinorrhea (82). Although the possibility of a physical obstruction (conductive olfactory loss) was initially considered a likely explanation for anosmia in COVID-19, it has now been questioned (82). The reason for this is that nearly 60% of the patients with anosmia do not have nasal congestion or rhinorrhea, and these patients lack any significant mucosal swelling of the nasal cleft or sinuses in imaging studies (66).

##### Damage to Supporting Cells in the Olfactory Epithelium

**Figure 3:**
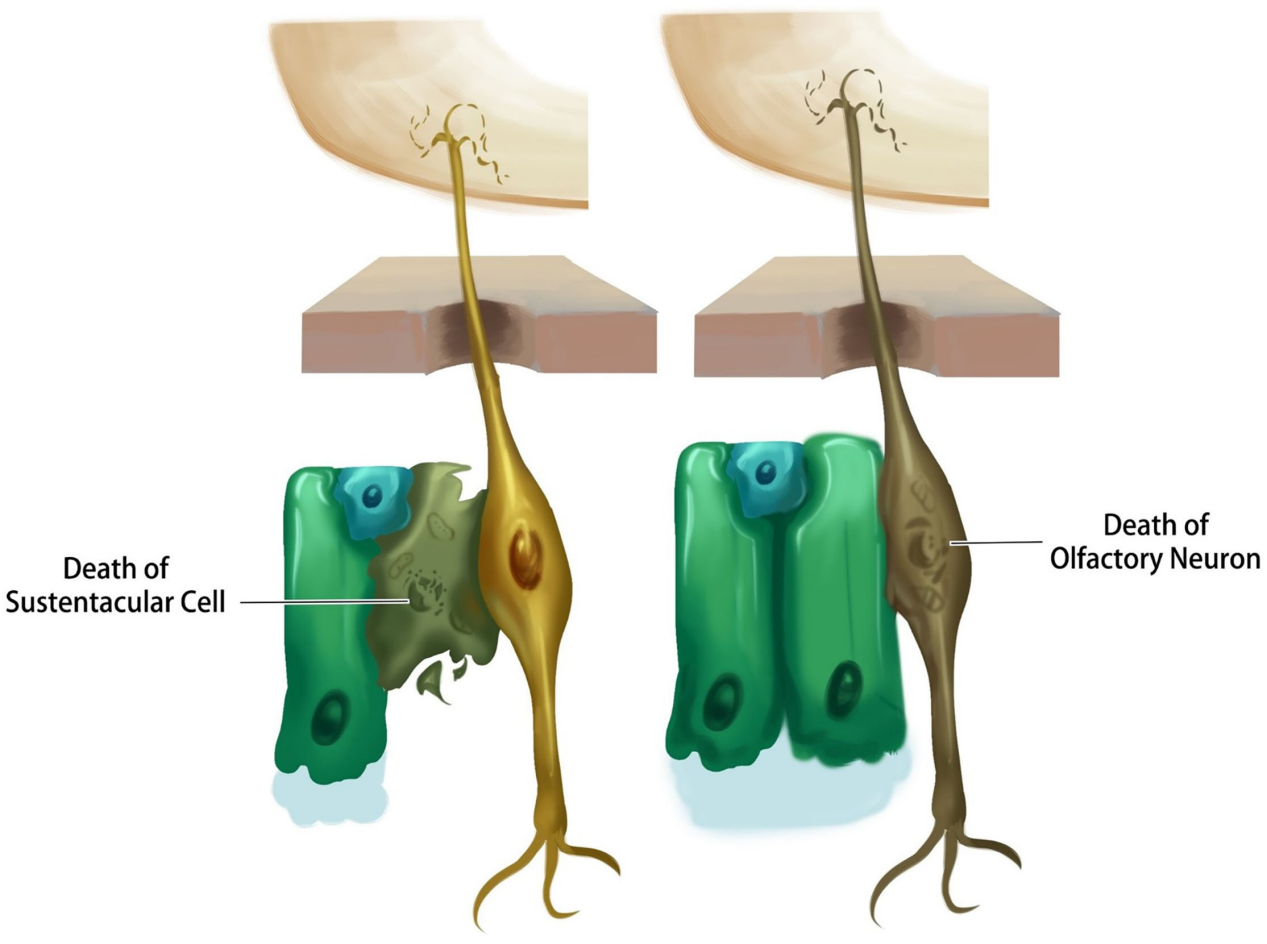
Schematic representation of different probable scenarios by which SARS-CoV-2 causes olfactory dysfunction.

Emerging evidence suggests that the loss of smell from coronavirus disease is a result of damage to the supporting cells rather than the neurons themselves. Post-mortem studies of people who had coronavirus disease found that the virus rarely reaches the brain (83). Recent evidence points to the possibility that the virus directly attacks the sustentacular cells of the olfactory epithelium, which have plenty of ACE2 receptors. Once the virus attaches to ACE2 receptors on sustentacular cells it induces cell death, leaving the sensory neurons vulnerable and deprived of nutrients and ultimately leading to olfactory dysfunction (84) (Figure 3).

##### Possible Death of Olfactory Neurons

The death of olfactory neurons following a viral attack is another explanation for the sensory neuron anosmia caused by coronavirus disease. To understand this, researchers have studied the expression of ACE2 receptors in the neuron. Based on *in-silico* data, it was shown that mature olfactory receptor neurons do not express ACE2 and TMPRSS2, and therefore are not likely to be infected by coronavirus (85). However, it was found that the other distinct cell types in the olfactory epithelium (sustentacular cells) have virus entry proteins, suggesting that olfactory neurons are not the primary target of the virus (86). Nonetheless, olfactory neuron death is reported in patients with prolonged cases of anosmia (Figure 3).

##### Possible Damage to the Olfactory Bulb and Cortex

Another possibility for olfactory dysfunction is brain infiltration by the virus, leading to changes in the olfactory centers that causes a reduced or complete loss of smell (87). At present, no study has demonstrated acute viral accumulation in olfactory receptor neurons or olfactory bulb neurons within the first 2 weeks of COVID-19 infection (88). There is also no evidence of coronavirus transmission to the brain through the olfactory route during the acute phase of anosmia. Brain tissue alterations observed on magnetic resonance imaging in COVID-19 patients were not consistent and may have been caused by virus-induced inflammation or by vascular transmission (89).

#### Pathophysiological Mechanisms of Gustatory Dysfunction

##### Role of Direct Infection on Taste Buds

Direct infection and subsequent death of taste cells is one possible mechanism for the transient loss of taste sensation from COVID-19 (90). Reduced secretion of neurotransmitters such as dopamine or serotonin by the infected taste cells or damage to stem cells by SARS-CoV-2 infection is another possibility (89).

##### Role of Renin–Angiotensin Aldosterone System

The local Renin–Angiotensin Aldosterone System (RAAS) is reported to be involved in the cleaving of gustatory molecules and the perception of taste (91). Viral binding to ACE2 could cause internalization of ACE2 and other components of the local RAAS system, leading to taste dysfunction. Since RAAS components are co-expressed with the salt and sweet receptors in the taste buds, modification of this system affects the host's perception of salty and sweet tastes (92).

##### Role of the Salivary Glands

Salivary gland infection by SARS-CoV-2 and the subsequent reduction in salivary flow is another possible mechanism for taste dysfunction in patients with COVID-19 (93). Altered saliva composition due to infection of salivary glands by SARS-CoV-2 is also a plausible cause for taste dysfunction (94).

##### Role of Sialic Acid

Sialic acid prevents the degradation of gustatory molecules that are bound to glycoproteins. The binding of SARS-CoV-2 to sialic acid residues in salivary mucin could cause gustatory molecule degradation and taste dysfunction (95).

##### Role of Interleukin-6

Taste dysfunction has been correlated with the levels of IL-6 in the blood of patients with COVID-19, and the recovery from taste dysfunction was correlated with a decline in the levels of IL-6. It has been hypothesized that IL-6 could exert a transient effect on taste cells rather than neuronal dysfunction, which would take longer to recover (96).

### SARS-CoV-2 Mutations, Variants, and Their Impact on Chemosensory Functions

Mutations in SARS-CoV-2 that resulted in new variants are another concern during this pandemic. Many SARS-CoV-2 mutations have been documented due to its high recombination rate, and are primarily noted in the spike protein, furin cleavage site, nucleocapsid protein, ORF genes (ORF1ab, ORF8, ORF1a, ORF3b, ORF6), NSP3, NSP12, NSP14, and the M gene (97). Butowt et al. (98) hypothesized that the D614G spike protein variant is a significant viral genetic factor that accelerates infection of the chemosensory epithelia. The D614G spike protein mutation in SARS-CoV-2 viruses may result in the increased prevalence of chemosensory dysfunction observed in East Asian and Western countries. The effects of mutations on disease phenotype are critical and may be due to the increased affinity of the G614 spike variant to ACE2 receptors or increased cell entry efficacy by spike protein stabilization and reduced cleavage. The G614 virus is capable of infecting the upper respiratory tract, which contains more nasal epithelial cells than lung epithelial cells. This contributes to a high viral load in the olfactory epithelium compared with the lower respiratory tract. A systematic review and meta-analysis performed by von Bartheld et al. (99) in South Asian populations reported a high prevalence of anosmia (31.8%) in populations infected predominantly with the G614 variant over the D614 virus strain (5.3%). It also concluded that the D614G mutation is a major contributing factor to the increased prevalence of anosmia in COVID-19. Following the observation of these mutations, both the WHO and the CDC independently classified multiple variants of SARS-CoV-2. The WHO classified the emerging variants into variants of concern (VOCs), variants of interest (VOIs), and variants under monitoring (VUM) (100).

### SARS-CoV-2 Variants of Concern

Variants of concern are variants that demonstrate evidence of enhanced transmissibility, more severe disease, reduction in neutralization by antibodies generated through natural infection or vaccination, reduced effectiveness of treatments or vaccines, or diagnostic detection failures. VOCs include the alpha, beta, gamma, and delta variants (101). Although these variants harbor the D614G spike protein mutation, the prevalence of olfactory dysfunction varies between them.

#### B.1.1.7 (Alpha)

B.1.1.7, also referred to as VOC 202012/01 or GRY, was the first COVID-19 variant of concern. It was identified in the United Kingdom in late December 2020 and has spread to 192 countries (102). There are 17 reported mutations in the viral genome of the B.1.1.7 variant, which includes 14 non-synonymous mutations and three deletion mutations. Eight mutations (Δ69–70 deletion, Δ144 deletion, N501Y, A570D, P681H, T716I, S982A, D1118H) are in the spike (S) protein (102). A REACT-1 study conducted by over 1 million people in the United Kingdom to identify symptoms predictive of COVID-19 in the community found that anosmia and an altered sense of taste were less predictive of B.1.1.7 compared with the wild type. In contrast, a sore throat and persistent cough were found to be more predictive of B.1.1.7 (103). Another report from Germany showed a negative association between anosmia/ageusia in patients infected with B.1.1.7 compared with the wild-type virus. A sore throat was more often a symptom in patients with the B.1.1.7 variant than the wild-type virus (54 vs. 42%, respectively) but anosmia or ageusia were less frequently associated with B.1.1.7 than the wild type (24 vs. 38%, respectively) (104). A sore throat, fatigue, and myalgias were more frequent symptoms of the alpha variant compared with olfactory dysfunction (105).

#### B.1.351 (Beta)

B.1.351, also referred to as the Beta variant or 20H501Y.V2, was initially detected in South Africa by the Center for the AIDS Programme of Research in December 2020 and has since spread to 141 countries. The beta variant emerged with 21 mutations, nine of which (L18F, D80A, D215G, R246I, K417N, E484K, N501Y, D614G, and A701V) were identified in the spike protein that enhance its strong binding to the ACE2 receptor and thereby increased its transmissibility (106). Literature mentioning chemosensory dysfunction caused by this variant is not available.

#### P.1 (Gamma)

The National Institute of Infectious Diseases in Japan first reported the P.1 variant in January 2021 during a routine airport screening of four travelers from Brazil (107). This variant includes 21 mutations, four synonymous mutations, 15 non-synonymous mutations, one insertion, and one deletion. A total of 10 mutations are in the spike protein, of which three (K417T, E484K, and N501Y) are in the receptor-binding domain (RBD), which increases its binding affinity to the ACE2 receptor (108). A prospective cohort study from Brazil showed a decreased incidence of anosmia/hyposmia and dysgeusia in Gamma variant cases (25 and 21%, respectively) compared with non-VOCs (46 and 38%, respectively). However, in a multivariate analysis anosmia/hyposmia (OR = 0.304, adj *P* < 0.001) and dysgeusia (OR = 0.385, adj *P* = 0.011) were the only symptoms significantly associated with the Gamma variant. There was no association between symptoms and age or gender (109).

#### B.1.617.2 (Delta)

The B.1.617.2 initially identified in India in December 2020 that is responsible for the second COVID-19 wave in India rapidly spread to more than 163 countries. The Delta variant has 13 mutations of which two are in the RBD (E484Q and L452R) (110). The Delta virus is more contagious (40%−60%) than the B.1.1.7 variant and twice as transmissible as the original SARS-CoV-2 Wuhan strain (101). No literature has evaluated chemosensory dysfunction caused by this variant, SARS-CoV-2 VOIs and VUM.

### Clinical Relevance of Chemical Sense Disorder in the Management of Patients With COVID-19

Both olfactory and taste dysfunction significantly affect the quality of life of patients with COVID-19, even if other symptoms are not severe. Anosmia and dysgeusia are early clinical symptoms of COVID-19 and have a prognostic value (111). A report also suggests that the presence of anosmia is fundamental to the diagnosis of novel coronavirus infection and is important for classifying patients and making therapeutic decisions (111). Studies have found a strong association between anosmia and a positive COVID-19 test. It, therefore, serves as a preliminary tool for identifying and isolating suspected cases. Olfactory and gustatory functional impairment are important predictors of clinical outcomes. Relatively rapid recovery of olfactory and gustatory functions can indicate the resolution of viral infection (71). However, permanent loss of olfaction in COVID-19 patients is an emerging concern. Ways to support or manage these patients need to be identified.

### Long-Term Morbidity and Management of Chemical Sense Disorder in COVID-19

A retrospective questionnaire study by Nguyen et al. (112) reported persistent olfactory and taste dysfunction 7 months after the onset of symptoms in 24% patients, of whom 23.3% of patients had a complete olfactory loss and 11.5% patients had complete loss of gustatory sense. Persistent symptoms were more common in women (73.3%) than men (26.7%). In another study, Le Bon et al. (74) observed persistent olfactory dysfunction in 37% of patients and dysgeusia in 7% of patients 37 days of symptom onset. A multicenter study reported that 24.1% of patients did not subjectively recover their olfactory function and 15.3% did not objectively recover olfactory function even 60 days after symptom onset (100). Lu et al. (113) observed that the bilateral gray matter volume (GMV) of the central olfactory system was smaller in patients with persistent olfactory loss compared with those without in COVID-19 recovered patients. It was proposed that altered epithelial homeostasis secondary to COVID-19 induces dysbiosis and chronic inflammation. This causes increased exfoliation, and the reduced number of taste receptors could be attributed to persistent dysgeusia in patients with COVID-19 (114). Persistent olfactory and taste dysfunction significantly worsens patient wellbeing and may cause psychiatric disorders such as depression, anxiety, and cognitive impairment. Accurate diagnosis and management of long-term olfactory and taste dysfunction is, therefore, essential.

Different measures such as olfactory training, oral or intranasal glucocorticoids, and zinc have been used to treat anosmia associated with COVID-19. Olfactory training is a disease-specific intervention that consists of a set of odorants such as lemon, rose, cloves, and eucalyptus that are repeatedly sniffed for 20 s each two times a day for at least 3 months. It is thought that the regenerative ability and the neuroplastic potential of olfactory neurons are increased with repeated stimulation by clearly defined odorants (115). Denis et al. (116) demonstrated that olfactory training and visual stimulation for an average of 4 weeks (28 days) significantly improved the olfaction of patients who received training for more than 28 days (73.3%) compared to a group who trained for <28 days (59%). The subjective olfactory dysfunction of patients with post-COVID-19 anosmia was significantly improved in both the mometasone furoate nasal spray group and the olfactory training group by the end of the third week (117). Systemic administration of prednisone and nasal irrigation with betamethasone, ambroxol, and rinazine for 15 days significantly improved the olfactory function of COVID-19 patients (118). The olfactory and gustatory recovery time was significantly shorter in COVID-19 patients treated with zinc therapy compared with controls (119). Triamcinolone oral paste significantly improved the gustatory dysfunction of patients with COVID-19 after 5 days of treatment (120).

## Conclusions and Future Research Directions

The cardinal early symptoms of coronavirus disease are the loss of smell and taste. The pooled prevalence of olfactory and taste dysfunction from coronavirus disease was 45.69 and 45.70%, respectively. The prevalence of olfactory dysfunction was 55.44, 56.05, 50.42, 28.83, and 26.50% in Europe, the Americas, the Middle East, Asia, and Africa, respectively. Similarly, the prevalence of gustatory dysfunction was 58.60, 52.53, 23.23, 27.19, and 22.10% in Europe, the Americas, the Middle East, Asia, and Africa, respectively. Several mechanisms may contribute to the loss of smell and taste. Many factors can be involved, and there may be a possible combined effect that finally leads to anosmia or dysgeusia. Strong evidence suggests that sensory neurons are not the primary target of the coronavirus, but rather that the supporting cells are primarily involved in the cascade of events that lead to anosmia. Viral cytotoxicity to the taste buds, direct viral invasion of the gustatory nerves, altered RAAS system, and increased pro-inflammatory cytokine expression were also possible contributors to dysgeusia from coronavirus disease. Further studies are required to understand the impact of coronavirus on chemosensory function. Future research needs to focus on how long COVID-19 affects sensations such as olfaction and gustation, and what could be done to effectively treat it. It is also important to understand the mechanisms behind the slow return or permanent loss of these senses even after the other symptoms of COVID-19 subside and the patient tests negative. Further studies may provide clarity on these poorly understood phenomena. Moreover, different variants of coronaviruses and their role in modulating chemosensory function is an emerging global concern. The primary reason for this is that the olfactory neurons are directly connected to the CNS, and if the newer variants get into the olfactory neurons easily, the virus can rapidly invade the brain through this pathway. This may further increase the chances that patients with COVID-19 will develop neurologic abnormalities or symptoms. Although this seems to be an unlikely possibility, we cannot completely exclude this as emerging evidence identifies the presence of viruses in the brains of patients with chronic anosmia and other symptoms. The mechanisms that underlie these findings are poorly understood. Further analysis is warranted to discern the pathophysiology of these neurologic disorders.

## Author Contributions

SNN conceived the manuscript, compiled various sections, and edited the final version of the manuscript. SNN, PS, SP, AB, and THM wrote different sections of the paper. YWL drawn figures and revised the paper. All authors contributed to the article and approved the submitted version.

## Conflict of Interest

The authors declare that the research was conducted in the absence of any commercial or financial relationships that could be construed as a potential conflict of interest.

## Publisher's Note

All claims expressed in this article are solely those of the authors and do not necessarily represent those of their affiliated organizations, or those of the publisher, the editors and the reviewers. Any product that may be evaluated in this article, or claim that may be made by its manufacturer, is not guaranteed or endorsed by the publisher.
